# Physiology and Plasticity of Interhemispheric Connections

**DOI:** 10.1155/2013/176183

**Published:** 2013-05-19

**Authors:** Matteo Caleo, Giorgio M. Innocenti, Maurice Ptito

**Affiliations:** ^1^CNR Neuroscience Institute, Via G. Moruzzi 1, 56124 Pisa, Italy; ^2^Department of Neuroscience, Karolinska Institutet, Retzius väg 8, SE-171 77 Stockholm, Sweden; ^3^École d'Optométrie, Université de Montréal, Montréal, QC, Canada H3T 1P1; ^4^Brain Lab, Department of Neuroscience and Pharmacology, Faculty of Health Sciences, University of Copenhagen, 2200 Copenhagen, Denmark

The corpus callosum (CC for aficionados) is the largest fiber bundle in the brain and establishes connections between the hemispheres, and predominantly, but not solely, between the cortical areas. Functionally mysterious for a long time, it shared with the pineal gland the honor of being considered the site of the soul [1]. The work on split brain in animals and humans performed by Gazzaniga, Mayer, Trevarthen, and Sperry, motivating the Nobel Prize to the latter, brought the CC on the stage of forefront international research. In spite of this, several questions remained open. What precisely is the function of the CC? What are the cellular and molecular mechanisms that persuade a set of cortical axons to take a route to the contralateral hemisphere? Which signals are responsible for the topography of callosal connections? Is the CC modifiable (plastic) in the adult brain? These questions are addressed in many recent studies and are represented by the papers collected in this issue. 

M. Fabri and G. Polonara provide a functional map of callosal topography by charting the BOLD signal evoked in callosal axons by taste, tactile, auditory, and visual stimuli and by motor tasks. This approach is at the frontier of what is usually obtained from BOLD signals. It provides results that are compatible with what is predicted by anatomy in the case of axons originating from primary areas, but it also shows activations that could not have been predicted from anatomy, probably due to axons originating in multisensory areas.

K. E. Schmidt finds that, in the visual cortex, CC connections have a multiplicative shift of the responses and this is an interesting finding that goes beyond the old debate of whether callosal connections are excitatory or inhibitory. The finding is placed within the frame of the historical question of the general nature of callosal connections. Hubel and Wiesel [2] were the first to propose that callosal connections are akin to intracortical connections, which have been “stretched” between the hemispheres. They wrote “*…a special set of connections exists for dealing with the midline representation of the visual fields. These fibers might be expected to serve the same functions as intracortical fibers linking cells with receptive fields clustered in other, more outlying parts of the visual fields.*” and the same concept was taken up some more times by others. The importance of the issue cannot be overemphasized. If indeed CC connections are like lateral or other intrahemispheric connections, they provide a general and advantageous model for the study of cortical connectivity. Callosal axons can be sectioned (as in the work quoted above) or reversibly inactivated far more easily and cleanly than most other lateral or intrahemispheric connections. Callosal axons can be studied in isolation at the cellular and molecular levels. Pathological alterations of callosal connections can herald conditions of more general cortical misconnectivity (discussed in [3]). If the nature of CC connections is indeed as reiterated by Schmidt, most of the papers collected in this issue can be read in a broader and fundamental framework.

V. Beaulé et al. focus on the role of CC connections in disentangling bilateral manual movements. From juvenile, to adult, to pathological conditions, the degrees of manual independence are differently modulated and this may be due to inhibitory action of callosal connections. Interestingly, inhibition between the hemispheres has been repeatedly reported for the motor functions, particularly in man, although it has been observed in the visual cortex as well, where it seems to be quickly overridden by the excitatory interactions [4].

Over the last 30 years, developmental work on the CC has focused on three main themes: (i) the molecular mechanisms of axonal guidance between the hemispheres, (ii) the establishment of topographical connections, and (iii) the role of activity in the development of the connections. M. Nishikimi et al. review the first of the above themes, with special attention to the midline structures and neighboring axons. They also describe alterations in these navigational mechanisms that result in callosal dysgenesis in humans and mice. Y. Tagawa and T. Hirano review the last of the above issues and provide information on the molecular mechanisms by which spontaneous activity sculpts callosal projections. They conclude that both presynaptic and postsynaptic neuronal activities are critically involved in callosal axon development, and discuss the intracellular signaling pathways that work downstream of neuronal firing.

It may be added that the overproduction and elimination of axons in development are central to the second of the themes above and continue to provide testable hypotheses on the nature of developmental plasticity of cortical connectivity [5]. Also, not only the topography of the connections but also the callosal axons themselves differentiate in development with axons from different areas acquiring different diameters and lengths, and therefore, presumably, generating specific conduction delays between the hemispheres [6].

Noninvasive structural and functional imaging techniques are taking an increasingly large share of brain studies, but this raises the question of how novel and more traditional, firmly established methodologies map onto each other. The CC is practically unavoidable in non-invasive structural studies, and, therefore, it can provide some general answers because of its central position in the brain, its relative “simplicity” and the amount of anatomical and functional information available. J. F. Olavarria et al. relate the critical period of callosal development, as defined by the reorganization of visual callosal connections caused by early enucleation, to the development of water diffusion parameters. This is important new information that complements the view that callosal plasticity relates to axonal maturation and differentiation. M. G. Knyazeva places callosal maturation as estimated by MRI and coherence EEG analysis, within the context of excitatory and inhibitory interactions between the hemispheres. P. Mathew et al. report data in preterm infants showing a relation between motor-specific scores and fractional anisotropy of anterior midbody of CC, the region where axons interconnecting motor areas course. Finally N. Takeuchi et al. introduce the concept of adult CC plasticity that might be elicited by trans-cranial stimulation in humans. They also discuss the use of brain stimulation techniques as a possible rehabilitation strategy to reinstate interhemispheric balance in patients with stroke.
